# Association of Bone Turnover Markers and Gla Rich Protein with Pelvic Calcification Severity and Clinical Outcomes in Kidney Transplant Recipients: A Prospective Single-Center Study

**DOI:** 10.3390/jcm15155982

**Published:** 2026-07-31

**Authors:** Iva Žuža, Antun Gršković, Slavica Kovačić, Ivan Brumini, Mate Kutlić, Robert Đurić, Nataša Katalinić, Ante Jakšić, Iva Bukša, Martina Mavrinac, Tanja Ćelić, Sanjin Rački, Lidija Orlić, Vesna Šupak Smolčić, Dean Markić

**Affiliations:** 1 Department of Radiology, Clinical Hospital Center Rijeka, 51000 Rijeka, Croatia; iva.zuza@medri.uniri.hr (I.Ž.); slavica.kovacic@medri.uniri.hr (S.K.); ivan.brumini@medri.uniri.hr (I.B.); 2 Faculty of Medicine, University of Rijeka, 51000 Rijeka, Croatia; antun.grskovic@medri.uniri.hr (A.G.); mate.kutlic@medri.uniri.hr (M.K.); robert.duric@medri.uniri.hr (R.Đ.); natasa.katalinic@medri.uniri.hr (N.K.); iva.buksa@medri.uniri.hr (I.B.); sanjin.racki@medri.uniri.hr (S.R.); lidija.orlic@medri.uniri.hr (L.O.); vesna.supak.smolcic@medri.uniri.hr (V.Š.S.); 3 Department of Urology, Clinical Hospital Center Rijeka, 51000 Rijeka, Croatia; 4 Clinical Institute of Transfusion Medicine, Clinical Hospital Center Rijeka, 51000 Rijeka, Croatia; 5 Faculty of Educational Sciences, University of Pula, 52100 Pula, Croatia; martina.mavrinac@unipu.hr; 6 Department of Nephrology, Dialysis and Transplantation, Clinical Hospital Center Rijeka, 51000 Rijeka, Croatia; 7 Clinical Department of Laboratory Diagnostics, Clinical Hospital Center Rijeka, 51000 Rijeka, Croatia

**Keywords:** kidney transplantation, vascular calcification, chronic kidney disease–mineral and bone disorder, osteoprotegerin, receptor activator of nuclear factor kappa-B ligand, Gla-rich protein, biomarkers, computed tomography

## Abstract

**Background**: Chronic kidney disease–mineral and bone disorder contributes to vascular calcification in kidney transplant recipients. However, the relationship between circulating bone turnover markers, Gla-rich protein (GRP), pelvic arterial calcification, and post-transplant outcomes remains uncertain. **Methods**: In this prospective single-centre study, 79 kidney transplant recipients underwent pre-transplant assessment of serum calcium, phosphate, alkaline phosphatase, parathyroid hormone, osteoprotegerin (OPG), receptor activator of nuclear factor kappa-B ligand (RANKL), and GRP. Pelvic arterial calcification was quantified using a validated CT-based scoring system. Associations between biomarkers, pelvic calcification severity, graft function, graft survival, patient survival, and major adverse cardiovascular events were evaluated. **Results**: Serum calcium was associated with serum creatinine (*p* = 0.039), and serum phosphate was associated with MAG-3 clearance (*p* = 0.009). OPG concentrations were significantly higher in patients receiving haemodialysis than in those receiving peritoneal dialysis (*p* = 0.021). No significant associations were observed between pelvic arterial calcification severity and circulating OPG, RANKL, or GRP concentrations. Furthermore, in univariable Cox proportional hazards models, none of the investigated biomarkers was significantly associated with graft or patient survival. **Conclusions**: Circulating OPG, RANKL, and GRP were not associated with pelvic arterial calcification, graft or patient survival. Larger multicentre studies with longer follow-up are warranted to clarify the prognostic value of these biomarkers.

## 1. Introduction

Changes in mineral and bone metabolism occur early during chronic kidney disease (CKD), typically when the glomerular filtration rate (GFR) declines below 60 mL/min/1.73 m^2^. These alterations are collectively referred to as chronic kidney disease–mineral and bone disorder (CKD-MBD), a systemic disorder characterised by abnormalities in calcium, phosphate, parathyroid hormone (PTH), and vitamin D metabolism, disturbances in bone turnover, and the development of vascular calcification (VC) [1]. CKD-MBD also encompasses abnormalities in bone mineralisation and bone volume and is recognised as one of the major contributors to the increased cardiovascular morbidity and mortality observed in patients with CKD [1,2].

Bone turnover is a fundamental component of skeletal homeostasis and is maintained through a tightly regulated balance between bone formation and bone resorption. In clinical practice, conventional biochemical markers, including serum calcium, phosphate, PTH, and alkaline phosphatase (AP), remain the cornerstone of bone turnover assessment. More recently, novel biomarkers involved in bone remodelling, such as osteoprotegerin (OPG) and receptor activator of nuclear factor kappa-B ligand (RANKL), have attracted increasing attention because of their potential roles in both bone metabolism and VC [3,4,5].

The receptor activator of nuclear factor kappa-B (RANK)/RANKL/OPG signalling pathway is one of the principal regulators of bone remodelling. Binding of RANKL to its receptor stimulates osteoclast differentiation and activation, whereas OPG acts as a soluble decoy receptor that inhibits this interaction, thereby suppressing osteoclastogenesis and bone resorption [5]. Beyond its physiological role in bone metabolism, the RANK/RANKL/OPG axis has also been implicated in vascular remodelling, endothelial dysfunction, inflammation, and arterial calcification. However, clinical studies evaluating circulating RANKL and OPG concentrations in CKD have yielded conflicting results. Serum RANKL concentrations have been reported to be either increased or unchanged at different stages of CKD compared with healthy individuals and generally decrease after the initiation of maintenance haemodialysis (HD) [6]. In contrast, circulating OPG concentrations progressively increase with declining kidney function and may normalise following successful kidney transplantation (KT) [7,8]. Furthermore, Nitta et al. demonstrated a significant positive correlation between serum OPG concentrations and the aortic calcification index in patients undergoing HD, suggesting that OPG may reflect the severity of VC in this population [9].

Gla-rich protein (GRP) is a vitamin K-dependent extracellular matrix protein that has recently emerged as a potent endogenous inhibitor of VC. Experimental studies have shown that GRP inhibits calcium-phosphate crystal formation and modulates the osteogenic differentiation of vascular smooth muscle cells. Clinical evidence indicates that circulating GRP concentrations progressively decline with advancing CKD stages in patients with diabetes mellitus and are associated with increasing VC, suggesting that GRP may represent an early biomarker of vascular injury in CKD [10].

A strong association between CKD-MBD, VC, and cardiovascular disease has been consistently demonstrated [11]. VC of the aortoiliac arteries is of particular clinical importance in kidney transplant candidates because these vessels are routinely used for vascular anastomosis during graft implantation. Consequently, severe calcification may increase surgical complexity, influence the choice of implantation site, and adversely affect post-transplant outcomes [12,13,14,15,16,17,18,19,20,21,22,23,24].

Previous studies investigating the prognostic significance of aortoiliac calcification in KT have reported conflicting findings. Aalten et al. observed no significant differences in one-year graft or patient survival between recipients with palpable iliac artery calcification and those without calcification (94% vs. 97% for graft survival and 97% vs. 98% for patient survival, respectively) [21]. Similarly, Werlin et al. found no significant differences in graft or patient survival among recipients with different CT-based iliac artery calcification scores [17]. In contrast, Davis et al. reported significantly lower three-year survival rates among recipients with iliac artery calcification than among those without calcification (94% vs. 87%) [16], while Disthabanchong et al. identified VC as an independent predictor of mortality in kidney transplant recipients [23].

Our group has previously applied the CT-based iliac artery calcification scoring system proposed by Davis et al. to evaluate pelvic vascular calcification in kidney transplant candidates [16,20,24]. In our initial study, recipients with a pelvic calcification score (PCS) greater than 3 had significantly shorter graft and patient survival than recipients with lower PCS values [20]. More recently, we confirmed that increasing pelvic arterial calcification severity was associated with inferior graft and patient survival, supporting the prognostic value of CT-based pelvic vascular calcification assessment before KT [24].

Despite increasing interest in bone remodelling biomarkers, relatively few prospective studies have simultaneously evaluated conventional bone turnover markers together with circulating OPG, RANKL, and GRP in relation to CT-defined pelvic vascular calcification and post-transplant clinical outcomes. Therefore, the aim of this prospective study was to investigate the associations between circulating bone turnover markers and GRP, pelvic vascular calcification, and clinical outcomes in kidney transplant recipients.

## 2. Materials and Methods

### 2.1. Study Population and Clinical Outcomes

**Figure 1:**
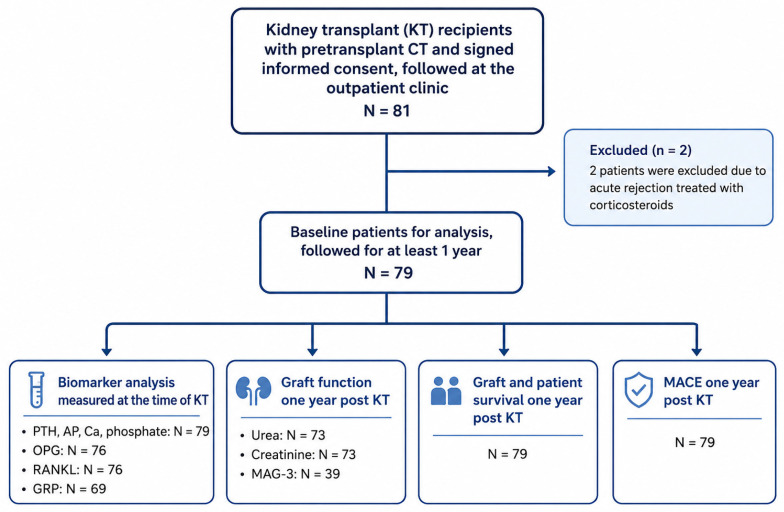
Study flow chart. Total number of patients included in the study with the number of patients according to serum biomarker analysis and clinical outcomes. Abbreviations: CT—computed tomography, PTH—parathyroid hormone, AP—alkaline phosphatase, Ca—calcium, GRP—Gla rich protein, OPG—osteoprotegerin, RANKL—receptor activator of nuclear factor kappa-B ligand, MAG-3—Technetium-99m mercaptoacetyltriglycine, MACE—major adverse cardiovascular events.

This prospective single-centre study included 79 consecutive patients who underwent KT at the Clinical Hospital Centre Rijeka, Croatia, between 21 May 2021 and 31 August 2023 (Figure 1). Details of our cohort, including demographic, clinical and post-transplant outcome data, have been presented in our previously published article [24]. The novelty of this study lies in the investigation of circulatory biomarkers in relation to pelvic vascular calcifications and post-transplant outcomes. These data are published for the first time.

Demographic and clinical data were retrieved from the institutional electronic medical records and included age, sex, body mass index (BMI), primary kidney disease, comorbidities, type and duration of renal replacement therapy, graft survival, patient survival, and the occurrence of major adverse cardiovascular events (MACE).

Patients were followed regularly at our transplant outpatient clinic for 12–36 months after KT. Clinical outcomes were evaluated one year after KT and included graft function, graft survival, patient survival, and the incidence of MACE. Graft function was assessed using serum creatinine, serum urea, and technetium-99m mercaptoacetyltriglycine (99mTc-MAG3) renal scintigraphy clearance. MAG-3 measurement one year after KT was not performed in all patients, mainly because some patients are not routinely referred to renal scintigraphy, and in some patients graft biopsy was performed as a better diagnostic modality.

Major adverse cardiovascular events were defined as myocardial infarction, cerebrovascular events, cardiovascular death, unstable angina requiring hospitalisation, peripheral arterial disease requiring revascularisation, heart failure requiring hospitalisation, or deep venous thrombosis (extended MACE definition).

Patients who experienced acute rejection episodes requiring corticosteroid treatment were excluded from the study. Post-transplant events were confirmed using clinical or imaging tools. We defined graft loss as the patient’s return to dialysis (either peritoneal dialysis or haemodialysis), surgical removal of the graft, or the patient’s death with a functioning organ. Graft survival was not death-censored; consequently, in the graft survival analysis, patients who died with a functioning graft were included among those who lost the kidney.

Our primary biomarker was GRP, which, prior to this study, had not been investigated in KT recipients. The main hypothesis is that KT recipients with higher serum GRP levels have less severe VC and better post-transplant outcomes. The primary clinical outcome included graft and patient survival in subgroups of KT recipients stratified according to their serum GRP levels.

### 2.2. Assessment of Pelvic Arterial Calcification

Pelvic arterial calcification was assessed using computed tomography (CT), as previously described by our group [20,24]. Briefly, two experienced radiologists independently evaluated arterial calcification using the semiquantitative scoring system proposed by Davis et al. [16].

The scoring system evaluates three characteristics of calcification: morphology, circumferential involvement, and longitudinal extent. Calcifications were assessed bilaterally in the common iliac arteries (CIA) and external iliac arteries (EIA). For each arterial segment, a calcification score (CS) ranging from 0 to 11 was calculated by summing the individual component scores. The overall pelvic calcification score (PCS) was obtained by summing the scores of all four arterial segments, resulting in a total score ranging from 0 (no calcification) to 44 (severe bilateral calcification).

For subgroup analyses, patients were primarily classified into three predefined categories according to PCS: Group I (PCS 0–4), Group II (PCS 5–19), and Group III (PCS > 19), as described previously [24]. As a secondary classification, based on our previous study, we divided patients into those with PCS ≤ 3 and those with PCS > 3 [20].

All CT examinations were independently evaluated by radiologists who were blinded to the patients’ clinical characteristics and laboratory findings.

### 2.3. Measurement of Bone Turnover Markers and Gla-Rich Protein

Peripheral venous blood samples were collected immediately before KT.

Serum concentrations of conventional bone turnover markers, including calcium, phosphate, AP, and PTH, were determined using routine laboratory methods at the Clinical Hospital Centre Rijeka.

Serum OPG, RANKL, and GRP concentrations were measured at the Department of Anatomy, Faculty of Medicine, University of Rijeka. Blood samples intended for OPG, RANKL, and GRP analyses were centrifuged immediately after collection, and serum aliquots were stored at −80 °C until analysis. Serum concentrations of total GRP, OPG and RANKL were measured using commercially available enzyme-linked immunosorbent assay (ELISA) kits according to the manufacturers’ instructions (Bioassay Technology Laboratory, Shanghai Korain Biotech, Shanghai, China). The following assays were used: total GRP ELISA Kit, Cat. No. E6794Hu, standard curve range 20–6000 ng/L, sensitivity 12.69 ng/L, intra-assay coefficient of variation (CV) < 8%; OPG ELISA Kit, Cat. No. E1558Hu, standard curve range 0.05–15 ng/mL, sensitivity 0.023 ng/mL, intra-assay CV < 8%; and RANKL ELISA Kit, Cat. No. E0620Hu, standard curve range 2–600 pg/mL, sensitivity 1.23 pg/mL, intra-assay CV < 8%. The GRP assay quantifies total circulating GRP and does not differentiate between carboxylated and uncarboxylated GRP fractions.

All measurements were performed in duplicate, and the final concentration of each biomarker was calculated as the arithmetic mean of the two measurements. A small number of ELISA measurements were considered unsuccessful because the calculated concentrations fell outside the assay’s reliable quantification range. Under normal circumstances, these samples would have been reanalysed. However, repeat measurements were not possible because insufficient sample material remained. Therefore, these measurements were excluded from the biomarker-specific statistical analyses, resulting in 76 participants for OPG and RANKL analyses and 69 participants for GRP analyses.

For survival analyses, recipients were stratified into low- and high-concentration groups according to the median serum value of each biomarker.

### 2.4. Kidney Transplantation and Immunosuppressive Protocol

Kidney transplantation was performed through an extraperitoneal approach using a standardised surgical technique described previously [25]. Briefly, the donor renal artery was anastomosed to the recipient’s external iliac artery, and the donor renal vein was anastomosed to the recipient’s external iliac vein. When the external iliac vessels were unsuitable for vascular anastomosis, the common iliac vessels were used instead.

The standard immunosuppressive regimen consisted of basiliximab induction therapy followed by maintenance immunosuppression with tacrolimus, mycophenolate mofetil, and prednisone.

### 2.5. Statistical Analysis

Statistical analyses were performed using MedCalc Statistical Software version 23.5.2 (MedCalc Software Ltd., Ostend, Belgium) and JASP, version 0.19.1 (JASP Team, 2024).

The primary analysis evaluated the association between serum GRP concentration and the continuous pelvic calcification score using Spearman’s rank correlation. An a priori sample-size calculation was performed using G*Power version 3.1.9.7, assuming a two-sided α of 0.05, 80% power, and an expected correlation coefficient of |r| = 0.30, re-sulting in a required sample size of 84 participants. Because GRP measurements were available for 69 participants, the study had 80% power to detect correlations of approximately |r| ≥ 0.33. All other analyses were considered secondary or exploratory. Categorical variables are presented as frequencies and percentages, whereas continuous variables are presented as medians with interquartile ranges (IQRs) or 5th–95th percentiles, as appropriate. Comparisons between categorical variables were performed using Pearson’s chi-square test or Fisher’s exact test when expected cell counts were <5. The distribution of continuous variables was assessed using the Kolmogorov–Smirnov test. Continuous variables are presented as medians and interquartile ranges. Analyses were conducted using available-case data without imputation. Mann–Whitney U and Kruskal–Wallis tests were used for group comparisons, with Dunn’s post hoc tests and Holm adjustment where applicable. Spearman’s rank correlation was used for associations between continuous variables. Twelve-month graft and patient survival were analysed using Kaplan–Meier methods, with administrative censoring at 12 months and comparison by the log-rank test. For graphical presentation, biomarker concentrations were dichotomised at the median; the low-concentration group (≤median) was the reference category, and hazard ratios represent the high-concentration group relative to the low-concentration group. Univariable Cox proportional hazards models were used, and the proportional hazards assumption was assessed using Schoenfeld residuals. Because of the small number of events, no multivariable Cox models were fitted, and survival analyses were considered exploratory. Analyses were performed using available-case data, without imputation of missing values. Patients with missing values for a particular biomarker or outcome were excluded only from the corresponding analysis. All statistical tests were two-sided, and a *p* value < 0.05 was considered statistically significant.

## 3. Results

### 3.1. Patient Characteristics and PCS

A total of 79 patients who underwent KT at the Clinical Hospital Center Rijeka between 2021 and 2023 were enrolled in this study. Table 1 summarises the demographic and clinical characteristics of the study population, including their PCS values. The median age of the cohort was 59 years (range, 16–76 years), the median BMI was 26 kg/m^2^, the median duration of dialysis prior to KT was 24 months, and the median cold ischaemia time was 910 min. The most common cause of ESKD was chronic glomerulonephritis, and eight patients underwent a second KT.

### 3.2. Clinical Outcomes After Kidney Transplantation

Kidney function one year after KT is presented in Table 2. Overall one-year graft survival was 92.4%, with graft loss occurring in six patients (including four who died with functioning grafts). One-year patient survival was 95.0%; four patients died during the first post-transplant year, all with functioning grafts at the time of death. MACE within the first year after transplantation was recorded in 15 patients (18.9%). The most common event was unstable angina pectoris, which occurred in five patients, followed by peripheral arterial disease requiring revascularisation procedures in four patients.

### 3.3. Serum Levels of Bone Turnover Markers and GRP at the Time of Transplantation Regarding Dialysis Modality and Principal Kidney Disease

The medians of bone turnover markers and GRP at the time of transplantation were within the reference intervals, except for PTH, which remained elevated (Table 3). In our cohort, pre-transplant OPG levels were significantly higher in patients undergoing HD than in patients treated with peritoneal dialysis (PD) (Table 4). There was no association between the principal kidney disease and the pre-transplant levels of OPG, RANKL and GRP (Supplementary File, Table S1).

### 3.4. Correlation of PCS with Serum Levels of Bone Turnover Markers and GRP

Correlation analysis revealed no significant relationships between PCS, bone turnover markers, or GRP (Table 5).

### 3.5. PCS Groups and Serum Levels of Bone Turnover Markers and GRP

Serum levels of bone turnover markers and GRP did not differ between the PCS groups (Table 6).

### 3.6. Correlation of Bone Turnover Markers and GRP with Creatinine, MAG-3 Clearance and MACE

The correlations between bone turnover markers and GRP with serum creatinine levels, MAG-3 clearance and MACE are presented in Table 7. Significant correlations were observed between serum calcium levels and serum creatinine (*p* = 0.039) and between serum phosphorus levels and MAG-3 clearance (*p* = 0.009). No other statistically significant correlations were identified (all *p* > 0.05).

### 3.7. Conventional Biomarkers and Graft and Patient Survival

For survival analyses in relation to conventional biomarkers (AP, calcium, phosphate and PTH), KT recipients were stratified into low- and high-concentration groups according to the median serum value of each biomarker. No statistically significant differences in graft or patient survival were observed between the low- and high-concentration groups, and none of the conventional biomarkers was significantly associated with survival in the univariable Cox proportional hazards analyses. All data are presented in the Supplementary File, Figures S1–S8.

### 3.8. OPG Groups and Clinical Outcomes

**Figure 2:**
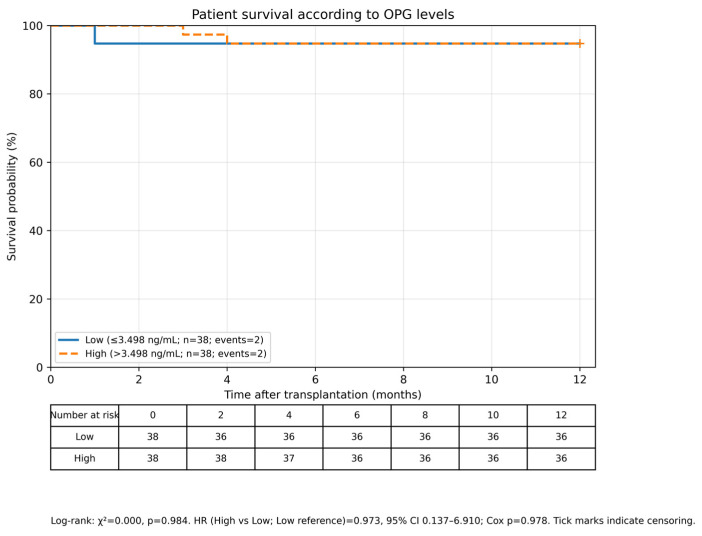
Kaplan-Meier survival curve for patient survival in relation to osteoprotegerin (OPG) levels (log-rank test: χ^2^ < 0.001, df = 1, *p* = 0.984).

**Figure 3:**
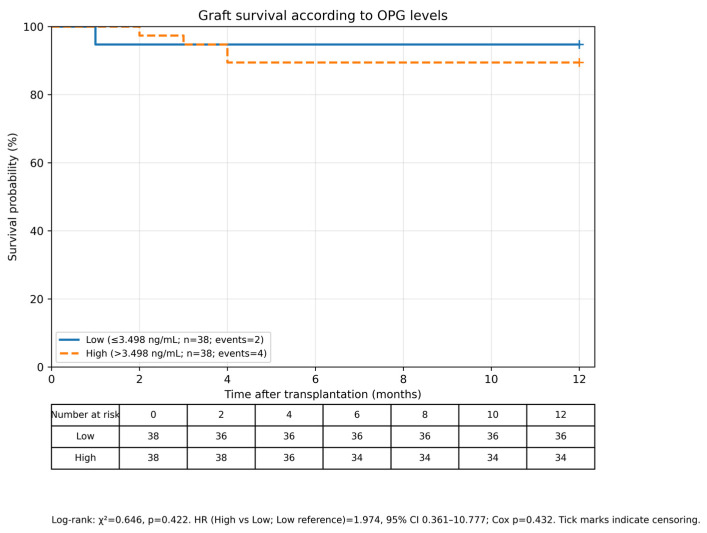
Kaplan-Meier survival curve for graft survival in relation to osteoprotegerin (OPG) levels (log-rank test: χ^2^ = 0.646, df = 1, *p* = 0.422).

Based on the median OPG concentration of 3.4984 ng/mL, patients were stratified into two groups: a low-OPG group (n = 38), defined as OPG ≤ 3.4984 ng/mL, and a high-OPG group (n = 38), defined as OPG > 3.4984 ng/mL. No statistically significant differences were observed between the groups and the investigated variables (Table 8). OPG concentrations were comparable between males and females, with median values of 3.60 ng/mL (IQR, 2.23–7.41) and 3.42 ng/mL (IQR, 1.90–6.32), respectively. Patients with a PCS score ≤3 exhibited lower OPG concentrations than those with a PCS score >3, with median values of 2.97 ng/mL (IQR, 2.18–5.93) and 4.05 ng/mL (IQR, 1.92–7.72), respectively. Patient and graft survival within 12 months did not differ significantly between the low- and high-OPG groups (Figure 2 and Figure 3).

### 3.9. RANKL Groups and Clinical Outcomes

**Figure 4:**
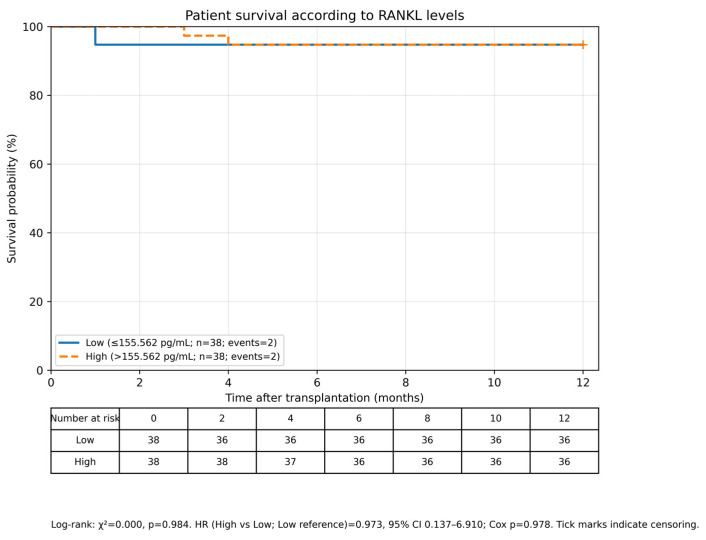
Kaplan-Meier survival curve for patient survival in relation to receptor activator of nuclear factor kappa-B ligand (RANKL) levels (log-rank test: χ^2^ = <0.001, df = 1, *p* = 0.984).

**Figure 5:**
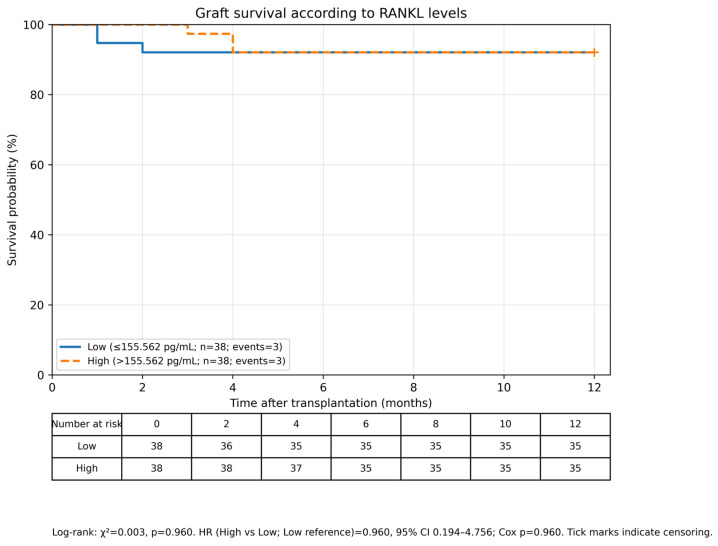
Kaplan-Meier survival curve for graft survival in relation to receptor activator of nuclear factor kappa-B ligand (RANKL) levels (log-rank test: χ^2^ = 0.003, df = 1, *p* = 0.96).

The median RANKL concentration in the study population was 155.56 pg/mL. Patients were stratified into two groups according to this median value: a low-RANKL group (≤155.56 pg/mL) and a high-RANKL group (>155.56 pg/mL), each comprising 38 patients. No statistically significant differences were observed between these groups in the investigated data (Table 9). RANKL concentrations were comparable between males and females, with median values of 155.56 pg/mL (IQR, 108–263) and 159.93 pg/mL (IQR, 93–261), respectively. Patients with a PCS score ≤3 had a median RANKL concentration of 163.64 pg/mL (IQR, 98–230), whereas those with a PCS score >3 had a median concentration of 152.60 pg/mL (IQR, 109–280). Patient and graft survival within 12 months did not differ significantly between the low- and high-RANKL groups (Figure 4 and Figure 5).

### 3.10. GRP Groups and Clinical Outcomes

**Figure 6:**
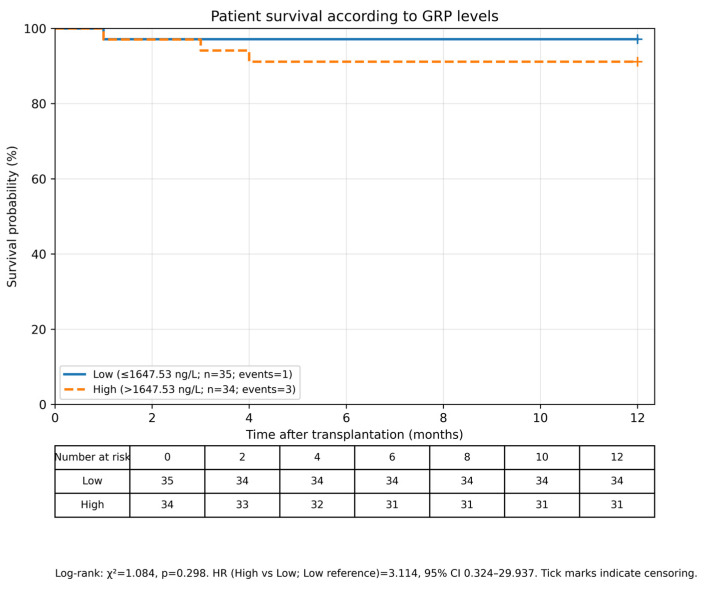
Kaplan-Meier survival curve for patient survival in relation to Gla-rich protein (GRP) levels (log-rank test: χ^2^ = 1.084, df = 1, *p* = 0.298).

**Figure 7:**
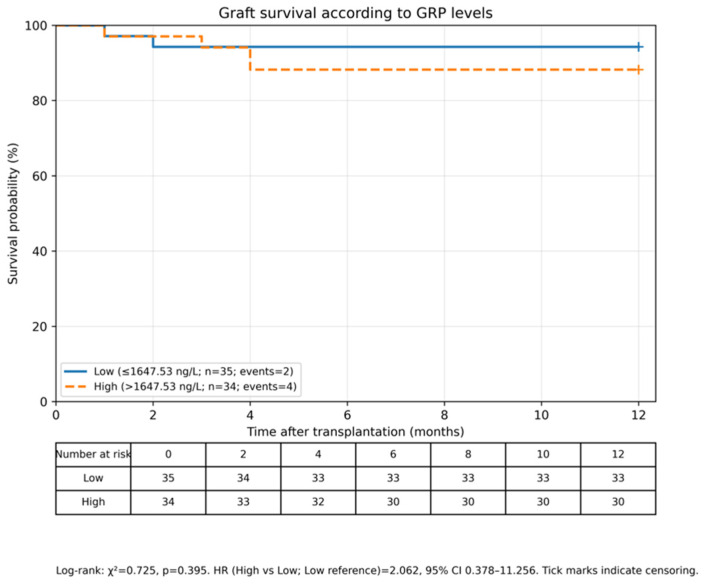
Kaplan-Meier survival curve for graft survival in relation to Gla-rich protein (GRP) levels (log-rank test: χ^2^ = 0.725, df = 1, *p* = 0.395).

Patients were divided into a low-GRP group (≤1647.53 ng/L; n = 35) and a high-GRP group (>1647.53 ng/L; n = 34) based on the median GRP concentration of 1647.53 ng/L. Baseline demographic, clinical, and laboratory characteristics were comparable between the groups, with no significant differences (Table 10). GRP concentrations were slightly higher in males than in females, with median values of 1830 ng/L (IQR, 822–3278) and 1480 ng/L (IQR, 968–2894), respectively. Patients with a PCS score ≤3 had lower GRP concentrations than those with a PCS score >3, with median values of 1502 ng/L (IQR, 984–2741) and 1785 ng/L (IQR, 808–3290), respectively. Patient and graft survival within 12 months did not differ significantly between the low- and high-GRP groups (Figure 6 and Figure 7).

## 4. Discussion

CKD is a major global health burden, with an estimated 697.5 million cases worldwide in 2017, corresponding to a global prevalence of 9.1% [26]. Between 1990 and 2017, CKD-associated mortality increased by 41.5%, and CKD is projected to become the fifth leading cause of death globally in the coming decades [26,27].

CKD-MBD, a common complication of CKD, is characterised by abnormalities in bone turnover, mineralisation, and skeletal growth and is frequently accompanied by vascular and soft tissue calcifications [1,28,29,30,31]. VC is now recognised as an active, highly regulated biological process that progresses with ageing [32]. Its prevalence and severity are particularly pronounced in patients with CKD and ESKD [33,34,35]. Although CKD-MBD plays a central role in the development of VC, it is not the sole mechanism responsible for its pathogenesis, as multiple metabolic, inflammatory, and cardiovascular factors also contribute to this complex process [33,34,35,36,37,38]. The osteogenic transformation of vascular smooth muscle cells results from an imbalance between promoters of VC, including hyperphosphataemia, hypercalcaemia, uraemia, oxidative stress, inflammation, bone morphogenetic proteins (BMP-2 and BMP-4), and excessive vitamin D activity, and endogenous inhibitors, such as GRP, OPG, fetuin-A, Klotho, vitamin K, pyrophosphate, magnesium, BMP-7, and physiological vitamin D signalling [34,39]. Several of these promoters and inhibitors are also involved in bone turnover, and their measurement may provide valuable insight into the complex mechanisms underlying both bone metabolism and VC. Their assessment may therefore improve the diagnosis of CKD-MBD and facilitate monitoring of VC progression. Consequently, circulating biomarkers have emerged as promising non-invasive tools that may partially complement bone biopsy, which remains the gold standard for assessing alterations in bone metabolism and enable a more individualised approach to the assessment and management of CKD-MBD. At present, no specific therapy has been approved for the treatment of VC [39,40].

Disturbances in mineral metabolism associated with CKD include alterations in serum calcium, phosphate, vitamin D, PTH, AP, fibroblast growth factor-23 (FGF-23), and Klotho concentrations [39]. In our study, median serum PTH concentrations were elevated in most patients, whereas calcium, phosphate, and AP levels remained within the reference ranges. This finding most likely reflects appropriate pre-transplant management of patients with ESKD, including dietary counselling and pharmacological treatment with phosphate binders, calcimimetic agents, vitamin D preparations, and calcium supplementation as part of CKD-MBD management. According to the Kidney Disease: Improving Global Outcomes (KDIGO) guidelines, serum PTH concentrations should be monitored regularly in patients with CKD and maintained within target ranges appropriate for the stage of CKD, generally between two and nine times the upper limit of normal [1]. These target ranges are intended to preserve appropriate bone turnover while minimising the risks associated with both low- and high-turnover bone disease. Nevertheless, elevated PTH concentrations were still observed in our cohort, and may contribute to the development of cardiovascular disease, soft tissue calcification, and an increased risk of fractures [39,41,42].

In addition to conventional bone turnover markers, we investigated emerging biomarkers involved in the regulation of bone metabolism and VC, including OPG, RANKL, and the VC inhibitor GRP. The RANK/RANKL/OPG signalling pathway plays an important role in bone remodelling, immune regulation, cancer development, and vascular biology. RANKL promotes osteoclast differentiation and activation, leading to bone resorption, whereas OPG acts as a decoy receptor that inhibits this signalling pathway [43].

Kosowski et al. demonstrated that serum OPG concentrations were significantly lower in healthy individuals than in patients with atherosclerosis and dyslipidemia (10.02 vs. 16.6 pmol/L), suggesting a close association between OPG and vascular disease [44]. Elevated circulating OPG concentrations have also been consistently reported in patients with ESKD [8,45,46]. In a study including 134 patients with CKD and 30 healthy controls, serum OPG concentrations increased progressively with declining kidney function and reached their highest values in patients undergoing HD [45]. Patients receiving HD had significantly higher OPG concentrations than predialysis patients (10.4 ± 4 vs. 7.03 ± 0.6 pmol/L). Kidney transplant recipients with a dialysis vintage longer than four years also exhibited higher OPG concentrations, whereas OPG concentrations decreased significantly one year after transplantation compared with both HD and predialysis patients and, in some cases, became lower than those observed in patients with stage 2 CKD [45]. Increased circulating OPG concentrations in HD patients have been attributed to reduced renal clearance, persistent high-turnover bone disease, and a compensatory response to ongoing vascular injury [46].

Consistent with these findings, serum OPG concentrations were higher in HD patients than in PD patients in our cohort. However, opposite findings have also been reported, with higher OPG concentrations observed in PD than in HD patients [47]. These discrepancies may reflect differences in patient characteristics, dialysis vintage, CKD-MBD severity, and study design, highlighting the need for further research to clarify OPG dynamics across different renal replacement therapy modalities.

VC remains one of the major cardiovascular complications of ESKD. Increasing age is a well-established risk factor for its development and progression. The median age of our study population was 59 years, which is comparable to that reported in previous kidney transplantation studies [15,16,17,20,24]. Consistent with previous reports, we also demonstrated that iliac artery calcification increased significantly with age [15,16,20,48]. Furthermore, in our cohort, recipients older than 55 years and those with hypertension, diabetes, or hyperlipidaemia had significantly higher PCS, supporting the contribution of traditional cardiovascular risk factors to VC.

In addition to these established risk factors, OPG, RANKL, and GRP have been proposed as potential circulating biomarkers of VC. Elevated OPG concentrations are generally considered to reflect vascular injury and endothelial dysfunction and may represent a compensatory response aimed at limiting VC [49].

In a cohort of 80 HD patients, higher serum OPG concentrations were associated with more severe VC and increased cardiovascular risk; however, they were not associated with calcification progression during follow-up. In the same study, serum RANKL concentrations showed no association with either the presence or progression of VC [49]. Similar findings have been reported by other investigators, who also failed to demonstrate a relationship between serum OPG concentrations and VC progression [49,50]. In contrast, a study including 47 patients with ESKD demonstrated significant associations between serum OPG concentrations and the progression of both atherosclerosis and VC, suggesting that OPG may represent a useful biomarker of cardiovascular complications and adverse clinical outcomes in dialysis patients [51]. Experimental studies further suggest that OPG acts primarily as an endogenous inhibitor and marker of VC rather than as a direct mediator of atherosclerosis [52]. In the present study, however, no significant association was observed between circulating OPG concentrations and the severity of VC.

Gupta et al. stratified kidney transplant recipients into three groups according to serum OPG concentrations and demonstrated that patients with OPG levels >4.2 pmol/L had lower estimated glomerular filtration rates and an increased risk of mortality [53]. Similarly, data from the ALERT study identified elevated serum OPG as an independent predictor of graft failure, doubling of serum creatinine, major cardiovascular events, cardiac mortality, and all-cause mortality [54]. In another study involving 173 kidney transplant recipients, patients in the highest OPG quartile had significantly higher all-cause and cardiovascular mortality than those with lower OPG concentrations; however, no association with graft survival was observed [55]. Comparable findings were reported in a German cohort of 600 kidney transplant recipients, in which elevated serum OPG concentrations were associated with patient survival but not graft survival [56]. Overall, these studies suggest that elevated circulating OPG is consistently associated with reduced patient survival, whereas its relationship with graft survival remains less consistent [53,54,55,56].

In contrast, a large Korean study including 1.018 kidney transplant recipients demonstrated that elevated OPG concentrations were associated with more extensive aortic and coronary artery calcification that persisted for up to five years after KT. Higher OPG levels were also independently associated with post-transplant cardiovascular events and graft survival, but not patient survival, highlighting the heterogeneity of findings across different populations [57].

In our cohort, there were no differences in OPG concentrations regarding VC and post-transplant outcomes. These findings support the concept that circulating OPG may primarily reflect a compensatory response to vascular injury rather than serve as a reliable predictor of VC or post-transplant outcomes [56]. Nevertheless, the precise biological role of OPG in VC after KT remains incompletely understood and warrants further mechanistic investigation.

The relationship between circulating RANKL concentrations and VC remains controversial [58,59,60,61,62,63]. In a study of 78 patients undergoing HD, serum RANKL concentrations were inversely correlated with coronary artery calcification scores [58]. Similarly, Wei et al. demonstrated that lower serum RANKL concentrations were associated with a higher risk of cardiovascular events in HD patients [59]. Conversely, several investigators failed to demonstrate significant associations between circulating RANKL concentrations and VC [60,61]. Furthermore, some studies have suggested that the OPG/RANKL ratio may better reflect the presence and progression of VC than either biomarker alone [48,58].

Consistent with these reports, no association between serum RANKL concentrations, VC and post-transplant outcomes was observed in our cohort. Taken together, these findings suggest that circulating RANKL alone has limited clinical utility as a biomarker of VC or post-transplant outcomes.

GRP has emerged as a potent endogenous inhibitor of VC through several complementary mechanisms, including inhibition of calcium-phosphate crystal maturation, antagonism of BMP-2-mediated osteogenic differentiation, and modulation of inflammatory signalling pathways [64,65,66]. In patients undergoing PD, circulating GRP concentrations were negatively correlated with serum phosphate, calcium, calcium-phosphate product, C-reactive protein, and VC, while showing a positive association with serum magnesium [67]. However, subsequent studies failed to confirm these findings or even reported positive associations between circulating GRP concentrations and VC [68,69]. In the present study there were no differences in OPG concentrations according to VC status or post-transplant outcomes.

To the best of our knowledge, this is the first prospective study to simultaneously evaluate circulating RANKL and GRP concentrations in relation to CT-defined pelvic VC and clinical outcomes in kidney transplant recipients. Larger multicentre studies with longer follow-up are required to better define the clinical significance of these biomarkers and their potential role in post-transplant cardiovascular risk stratification.

This study has several limitations. First, it was conducted at a single centre and included a relatively small cohort with a limited follow-up period, which may have reduced the statistical power to detect modest associations between circulating biomarkers and clinical outcomes. Future studies investigating biomarkers and VC in patients with ESKD should include larger cohorts, allowing adjustment for a greater number of potential confounding variables and thereby improving the precision and robustness of the findings. Second, bone turnover markers and GRP were measured only once, immediately before transplantation. Serial measurements after KT could provide additional insight into the temporal relationship between biomarker dynamics, VC progression, and long-term transplant outcomes. Third, there were only four patient deaths and six graft losses in our study; consequently, the survival analyses have limited statistical power. Also, the wide confidence intervals in the analysis of graft and patient survival in relation to different biomarker levels indicate that our study is statistically underpowered for the determination of survival endpoints, which is also a limitation of our study. Therefore, the conclusion that biomarkers are not predictive for survival outcomes should be treated with caution. Since the patients who developed acute rejection requiring corticosteroid treatment were excluded from the study, this represents a potential source of selection bias, particularly because graft function and graft survival are the study outcomes.

Another potential source of selection bias is the lack of MAG-3 data in almost half of the patients.

The principal strengths of this study include its prospective design and the simultaneous evaluation of conventional and novel bone turnover biomarkers, GRP, CT-defined VC, and post-transplant clinical outcomes. Biomarker concentrations were quantified using standardised ELISA assays, and VC was assessed using a validated CT-based scoring system. Furthermore, the inclusion of an unselected kidney transplant population, without restrictions based on age, dialysis duration, smoking status, comorbidities, or ethnicity, enhances the generalisability of our findings compared with studies applying more restrictive selection criteria [70].

## 5. Conclusions

In conclusion, this small, single-centre, prospective study demonstrated that conventional markers of mineral metabolism, as well as OPG, RANKL, and GRP, were not associated with CT-defined pelvic VC, graft, or patient survival. These findings suggest that, despite their established biological roles in bone remodelling and VC, the clinical utility of these emerging biomarkers for risk stratification in kidney transplant recipients remains limited. Since clinically relevant associations were not demonstrated in this cohort, larger multicentre prospective studies with longer follow-up are warranted to clarify their role in the pathophysiology of VC and their potential prognostic value in kidney transplant recipients.

## Figures and Tables

**Table 1:** Demographic and clinical characteristics of the patients, including pelvic calcification scores (N = 79).

Demographic and Clinical Data				Pelvic Calcifications Scores (PCS)	
	N	%	P^1^	Median (5–95 Percentiles)	P^2^
Age					
≤55	36	45.5	0.428	3 (0–21.8)	<0.001
>55	43	54.5		19 (0–36.7)	
Gender					
Female	29	36.7	0.018	12.5 (0–33.1)	0.354
Male	50	63.3		10 (0–36)	
Hypertension					
Yes	68	86.1	<0.001	13.5 (0–35)	0.017
No	11	13.9		4 (0–18.9)	
Diabetes					
Yes	25	31.6	0.001	24 (0–39.5)	<0.001
No	54	68.4		6 (0–25.8)	
Hyperlipidemia					
Yes	27	34.2	0.005	19 (3.85–37.2)	<0.001
No	52	65.8		6 (0–32.9)	
Hyperparathyroidism					
Yes	76	96.2	<0.001	11.5 (0–35)	0.747
No	3	3.8		18 (0–33)	
Principal disease					
Diabetes	9	11.4	0.017	24 (0–33)	0.002 *
Chronic glomerulonephritis	26	32.9		10 (0–35.6)	
Nephroangiosclerosis	16	20.3		25 (1.8–41.3)	
Polycystic kidney disease	10	12.7		5.5 (0.36)	
Others	18	22.8		3.5 (0–29.2)	
Renal replacement therapy					
Haemodialysis	52	65.8	<0.001	10.5 (0–36)	0.163
Peritoneal dialysis	19	24.1		13 (0–36.6)	
Without dialysis	8	10.1		19.5 (5–32)	
Donor origin					
Croatia	44	55.7	0.311	13.5 (0–36.6)	0.592
Other Eurotransplant states	35	44.3		11 (0–34.5)	
Donor type					
Donation after brain death	76	96.2	<0.001	12.5 (0–35)	0.450
Living-related	3	3.9		12	
Transplantation					
First	71	89.8	<0.001	13 (0–35)	0.051
Second	8	10.2		1.5 (0–22)	
MACE					
Yes	15	19	<0.001	19 (0–35)	0.128
No	64	81		10 (0–35.3)	

MACE-major adverse cardiovascular events; for P^1^ was used Chi-square test (χ^2^) and for P^2^ was used Mann-Whitney U test or Kruskal–Wallis tests for group comparisons, with Dunn’s post hoc tests and Holm adjustment where applicable * Dunn post hoc analysis: Pelvic calcification scores differed significantly according to the underlying kidney disease, H(4) = 17.43, *p* = 0.002, rank ε^2^ = 0.223. Dunn’s post hoc test with Holm correction showed significantly higher PCS values in patients with diabetes mellitus (*p* = 0.026) and nephroangiosclerosis (*p* = 0.006) compared with patients with other underlying kidney diseases. No other pairwise differences were statistically significant.

**Table 2:** Kidney function one year after kidney transplantation.

Variables	N	Median	5th–95th Percentiles	Reference Intervals
Urea (mmol/L)	73	8.25	4.58–14.7	2.8–8.3
Creatinine (μmol/L)	73	114.5	75–183	64–104
MAG-3 Clearance (mL/min/1.73 m^2^)	39	158	67–276	>200

**Table 3:** Values of bone turnover markers and Gla-rich protein at the time of kidney transplantation.

Bone Turnover Markers (Units)	N	Median	5th–95th Percentiles	Reference Intervals
Calcium (mmol/L)	79	2.3	2–2.7	2.14–2.53
Phosphate (mmol/L)	79	0.87	0.43–2.35	0.79–1.42
Alkaline phosphatase (IU/L)	79	78	38–165	60–142
PTH (pmol/L)	79	16.79	6.26–54	1.6–6.9
GRP (ng/L)	69	1647	333–6350	/
OPG (ng/mL)	76	3.49	0.73–14.77	/
RANKL (pg/mL)	76	156	56–604	/

PTH—parathyroid hormone, GRP—Gla-rich protein, OPG—osteoprotegerin, RANKL—receptor activator of nuclear factor kappa-B ligand.

**Table 4:** Values of Gla-rich protein, receptor activator of nuclear factor kappa-B ligand and osteoprotegerin between different dialysis modalities.

	N	GRP (ng/L) N = 69		N	RANKL (pg/mL) N = 76		N	OPG (ng/mL) N = 76	
Dialysis Modality		Median (IQR)	*p*		Median (IQR)	*p*		Median (IQR)	*P*
Hemodialysis	45	1831 (979–3377)	0.104	50	167 (123–314)	0.059	50	4.09 (2.43–8.4)	0.021 *
Peritoneal dialysis	16	1275 (631–1966)		18	109 (92–168)		18	1.86 (1.27–3.57)	
No dialysis	8	2071 (780–3792)		8	128 (105–266)		8	3.87 (2.5–7.66)	

IQR—interquartile range, GRP—Gla rich protein, OPG—osteoprotegerin, RANKL—receptor activator of nuclear factor kappa-B ligand; * Post–hoc Dunn test, patients on hemodialysis has significant higher OPG values compared to patients on peritoneal dialysis (*p* < 0.05).

**Table 5:** Correlation between pelvic calcification score, bone turnover markers and Gla-rich protein.

Bone Turnover Markers (Units)	Pelvic Calcification Score—Spearman Rank Correlation	
	r_s_	*P*
PTH (pmol/L)	−0.005	0.963
Calcium (mmol/L)	−0.15	0.200
Phosphate (mmol/L)	0.04	0.706
Alkaline phosphatase (IU/L)	0.08	0.485
GRP (ng/L)	−0.03	0.785
RANKL (pg/mL)	−0.006	0.956
OPG (ng/mL)	0.028	0.809

PTH—parathyroid hormone, GRP—Gla-rich protein, OPG—osteoprotegerin, RANKL—receptor activator of nuclear factor kappa-B ligand.

**Table 6:** Circulating levels of bone turnover markers and GRP across PCS groups.

	PCS Groups			
	1 (N = 27)	2 (N = 29)	3 (N = 23)	*P*
Variables	Median (5th–95th Percentile)			
Calcium (mmol/L)	2.35 (2.08–2.71)	2.29 (1.96–2.60)	2.27 (1.9–2.71)	0.240
Phosphate (mmol/L)	0.82 (0.40–2.44)	0.87 (0.51–2.35)	0.89 (0.42–1.89)	0.740
AP (IU/L)	73 (37–139)	85 (54–166)	81 (43–170)	0.099
PTH (pmol/L)	16.34 (8–57)	19.6 (6.09–50.7)	15.14 (5.4–68)	0.468
GRP (ng/L)	1558 (346–6413)	1647 (323–7004)	1952 (306–6039)	0.958
RANKL (pg/mL)	163 (53–630)	154 (59–533)	138 (46–591)	0.876
OPG (ng/mL)	3.4 (0.37–14.63)	4.1 (1.33–16.26)	3.52 (0.57–14.72)	0.944

AP—alkaline phosphatase, PTH—parathyroid hormone, GRP—Gla-rich protein, OPG—osteoprotegerin, RANKL—receptor activator of nuclear factor kappa-B ligand, (Kruskall-Wallis test).

**Table 7:** Associations of bone turnover markers and GRP with serum creatinine, MAG-3 clearance and major adverse cardiovascular events (MACE) one year after kidney transplantation (Spearman rank correlation-r_s)_.

Variables	r_s_/*p*	Creatinine	MAG 3 Clearance	MACE
PTH (pmol/L)	r_s_	0.010	−0.218	−0.173
	*p*	0.931	0.183	0.128
Calcium (mmol/L)	r_s_	0.241	−0.025	0.168
	*p*	0.039	0.877	0.139
Phosphate (mmol/L)	r_s_	−0.036	−0.415	−0.045
	*p*	0.762	0.009	0.692
AP (IU/L)	r_s_	−0.002	0.059	0.074
	*p*	0.984	0.719	0.515
GRP (ng/L)	r_s_	0.096	−0.148	0.069
	*p*	0.450	0.404	0.574
RANKL (pg/mL)	r_s_	0.187	−0.130	0.003
	*p*	0.119	0.444	0.979
OPG (ng/mL)	r_s_	0.213	−0.074	0.029
	*p*	0.074	0.659	0.801

AP—alkaline phosphatase, PTH—parathyroid hormone, GRP—Gla -rich protein, OPG—osteoprotegerin, RANKL—receptor activator of nuclear factor kappa-B ligand.

**Table 8:** Characteristics of kidney transplant patients based on circulating levels of osteoprotegerin (OPG).

		OPG (N = 76)			
Variables	All	Low OPG	High OPG	Test *	*p*
Age (years)	59 (47–67)	58.5 (46–66)	56.5 (51–67)	MW	0.596
Gender Male/Female	48/28	23/15	25/13	χ^2^	0.637
Time on dialysis (months)	24 (10.5–42)	24 (18–62)	18 (8–36)	MW	0.095
Urea (mmol/L)	8.3 (6.4–10.3)	7.6 (6.1–10.8)	8.4 (6.5–9.5)	MW	0.995
Creatinine (µmol/L)	114 (90–139)	106 (88–124)	118 (96.5–146)	MW	0.068
PCS ≤ 3/PCS > 3	22 (28.9%)/54 (71.1%)	13/25	9/29	χ^2^	0.315
MAG-3 (N = 39)	158 (119.5–201.5)	171 (136–201) N = 20	142 (116–202) N = 19	MW	0.599
MACE no/yes	62/14	32/6	30/8	χ^2^	0.557

Data are shown with Median and IQR or frequencies. * Mann-Whitney U test (MW), chi-square test (χ^2^).

**Table 9:** Characteristics of KT patients based on circulating levels of receptor activator of nuclear factor kappa-B ligand (RANKL).

	RANKL (N = 76)				
Variables	All	Low RANKL	High RANKL	Test *	*p*
Age (years)	59 (47–66.75)	59.5 (49–68)	57.5 (46–64)	MW	0.377
Gender Male/Female	48/28	24/14	24/14	χ^2^	1
Time on dialysis (months)	24 (10.5–42)	23 (8–48)	24 (12–36)	MW	0.499
Urea (mmol/L)	8.3 (6.4–10.3)	8.4 (6.2–11.8)	7.85 (6.4–9.4)	MW	0.434
Creatinine (µmol/L)	114 (90–139)	108 (90–124)	111 (91–147)	MW	0.160
PCS ≤ 3/PCS > 3	22/54	10/28	12/26	χ^2^	0.615
MAG-3 N = 39	158 (119.5–201.5)	154 (137–186) N = 18	160 (111–202) N = 21	MW	0.963
MACE no/yes	62/14	31/7	31/7	χ^2^	1

Median (IQR) and frequencies; * Mann-Whitney U test (MW) or chi-square test (χ^2^).

**Table 10:** Characteristics of kidney transplant patients based on circulating levels of Gla-rich protein (GRP).

		GRP (N = 69)			
Variables	All	Low GRP (N = 35)	High GRP (N = 34)	Test *	*p*
Age (years)	59 (47–66.75)	59 (46–66)	59 (47–69)	MW	0.801
Gender Male/Female	43/26	20/15	23/11	χ^2^	0.371
Time on dialysis (months)	24 (10.5–42)	24 (13–61)	20.5 (9–36)	MW	0.282
Urea (mmol/L)	8.3 (6.4–10.3)	8 (6.6–10.4)	8.3 (6.1–9.5)	MW	0.586
Creatinine (µmol/L)	114 (90–139)	108 (89–126)	116 (91–150)	MW	0.276
PCS ≤ 3/PCS > 3	19/50	11/24	8/26	χ^2^	0.466
MAG-3 N = 39	158 (119.5–201.5)	169 (153.5–201) N = 18	137.5 (101–203) N = 21	MW	0.427
MACE no/yes	56/13	30/5	26/8	χ^2^	0.3298

Median (IQR) and frequencies, * Mann-Whitney U test (MW) or chi-square test (χ^2^).

## Data Availability

The data presented in this study are available from the corresponding authors upon reasonable request. The data are not publicly available due to privacy and ethical restrictions related to patient confidentiality.

## Supplementary Materials

The following supporting information can be downloaded at: https://www.mdpi.com/article/10.3390/jcm15155982/s1, Table S1. Circulating levels of GRP, RANKL, and OPG according to principal kidney disease. Figure S1. Kaplan-Meier survival curve for graft survival in relation to serum level of alkaline phosphatase. Figure S2. Kaplan-Meier survival curve for patient survival in relation to serum level of alkaline phosphatase. Figure S3. Kaplan-Meier survival curve for graft survival in relation to serum level of calcium. Figure S4. Kaplan-Meier survival curve for patient survival in relation to serum level of calcium. Figure S5. Kaplan-Meier survival curve for graft survival in relation to serum level of phosphate. Figure S6. Kaplan-Meier survival curve for patient survival in relation to serum level of phosphate. Figure S7. Kaplan-Meier survival curve for graft survival in relation to serum level of parathyroid hormone (PTH). Figure S8. Kaplan-Meier survival curve for patient survival in relation to serum level of parathyroid hormone (PTH).

## Abbreviations

AP	Alkaline phosphatase
BMI	Body mass index
CIA	Common iliac artery
CKD	Chronic kidney disease
CKD-MBD	Chronic kidney disease–mineral and bone disorder
CS	Calcification score
CT	Computed tomography
CV	Coefficient of variation
EIA	External iliac artery
ELISA	Enzyme-linked immunosorbent assay
ESKD	End-stage kidney disease
GFR	Glomerular filtration rate
GRP	Gla-rich protein
HD	Hemodialysis
HR	Hazard ratio
IQR	Interquartile range
KT	Kidney transplantation
MACE	Major adverse cardiovascular events
MAG-3	Technetium-99m mercaptoacetyltriglycine
OPG	Osteoprotegerin
PCS	Pelvic calcification score
PD	Peritoneal dialysis
PTH	Parathyroid hormone
RANK	Receptor activator of nuclear factor kappa-B
RANKL	Receptor activator of nuclear factor kappa-B ligand
VC	Vascular calcification
