# Supplementary material for: Association of Bone Turnover Markers and Gla Rich Protein with Pelvic Calcification Severity and Clinical Outcomes in Kidney Transplant Recipients: A Prospective Single-Center Study
Source: J Clin Med. 2026 Jul 31;15(15):5982. doi: 10.3390/jcm15155982 (PMC13466340; doi:10.3390/jcm15155982)
Supplement: Supplementary file 1 [file jcm-15-05982-s001.zip › jcm-4445410-supplementary.pdf]

Table S1. Circulating levels of GRP, RANKL, and OPG according to principal kidney disease

Principal kidney disease	GRP (ng/L) N = 69			RANKL (pg/mL) N = 76			OPG (ng/mL) N = 76		
	N	Median (IQR)	p	N	Median (IQR)	p	N	Median (IQR)	**p**
Diabetes mellitus	7	939.85 (733.96–4312.16)	0.926	9	142.01 (95.93–205.69)	0.973	8	3.85 (1.89–7.61)	0.959
Chronic glomerulonephritis	21	1502.76 (1273.84–2759.79)		24	161.27 (107.85–232.40)		24	2.95 (1.79–6.38)	
Nephroangiosclerosis	14	1947.85 (1046.49–3487.85)		15	166.60 (85.68–307.08)		16	4.19 (1.85–7.91)	
Polycystic kidney disease	10	1731.84 (1033.02–2726.84)		10	151.61 (123.05–285.91)		10	3.52 (2.18–6.12)	
Other diseases	17	1647.53 (878.36–3144.50)		18	155.56 (105.70–299.16)		18	3.50 (2.38–8.07)	

Data are presented as median and interquartile range (IQR). Differences between groups were assessed using the Kruskal–Wallis test. GRP, Gla-rich protein; RANKL, receptor activator of nuclear factor kappa-B ligand; OPG, osteoprotegerin

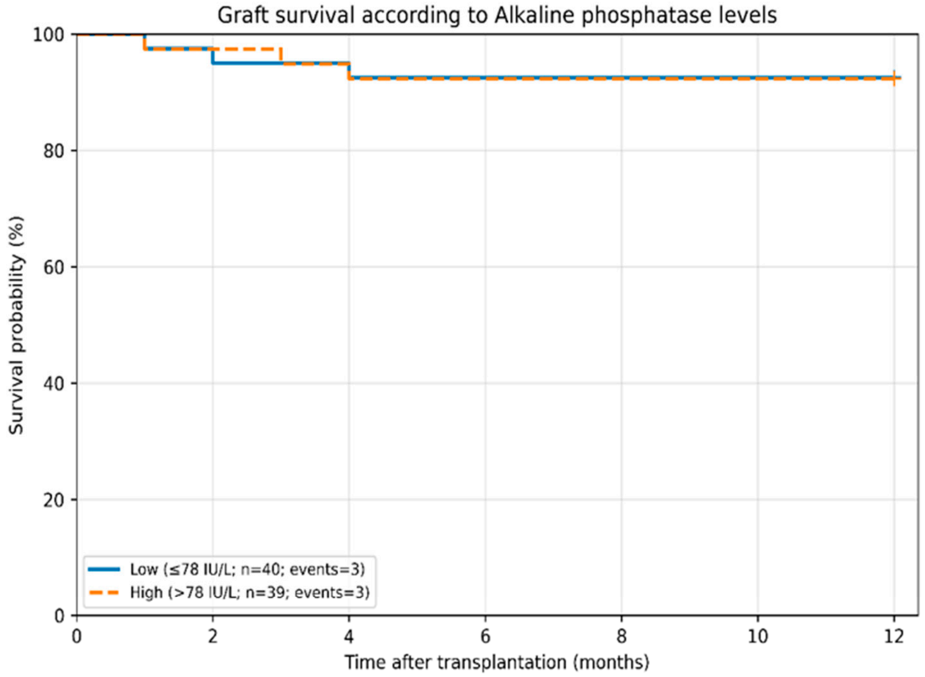

Number at risk	0	2	4	6	8	10	12
Low	40	39	38	37	37	37	37
High	39	38	37	36	36	36	36

Log-rank: χ^2=0.001, p=0.979. HR (High vs Low; Low reference)=1.022, 95% CI 0.206–5.064; Cox p=0.979. Tick marks indicate censoring; marks at 12 months overlap.

Figure S1. Kaplan-Meier survival curve for graft survival in relation to serum level of alkaline phosphatase.

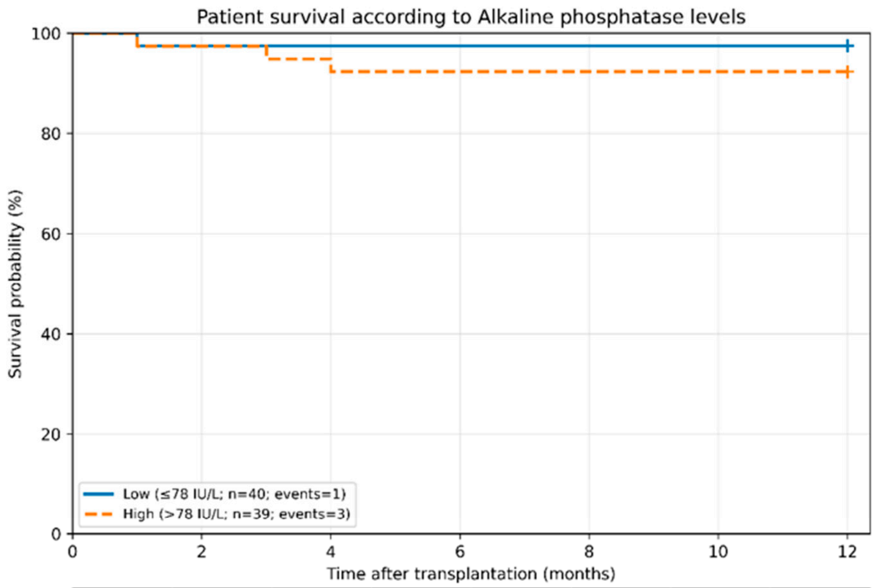

Number at risk	0	2	4	6	8	10	12
Low	40	39	39	39	39	39	39
High	39	38	37	36	36	36	36

Log-rank: χ^2=1.073, p=0.300. HR (High vs Low; Low reference)=3.099, 95% CI 0.322–29.792; Cox p=0.327. Tick marks indicate censoring; marks at 12 months overlap.

Figure S2. Kaplan-Meier survival curve for patient survival in relation to serum level of alkaline phosphatase.

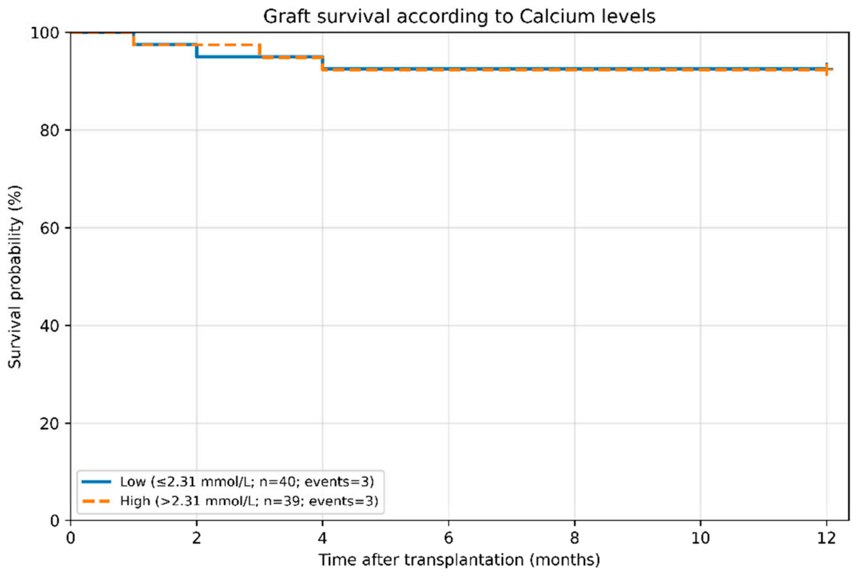

Number at risk	0	2	4	6	8	10	12
Low	40	39	38	37	37	37	37
High	39	38	37	36	36	36	36

Log-rank: χ^2=0.001, p=0.979. HR (High vs Low; Low reference)=1.022, 95% CI 0.206–5.064; Cox p=0.979. Tick marks indicate censoring; marks at 12 months overlap.

Figure S3. Kaplan-Meier survival curve for graft survival in relation to serum level of calcium.

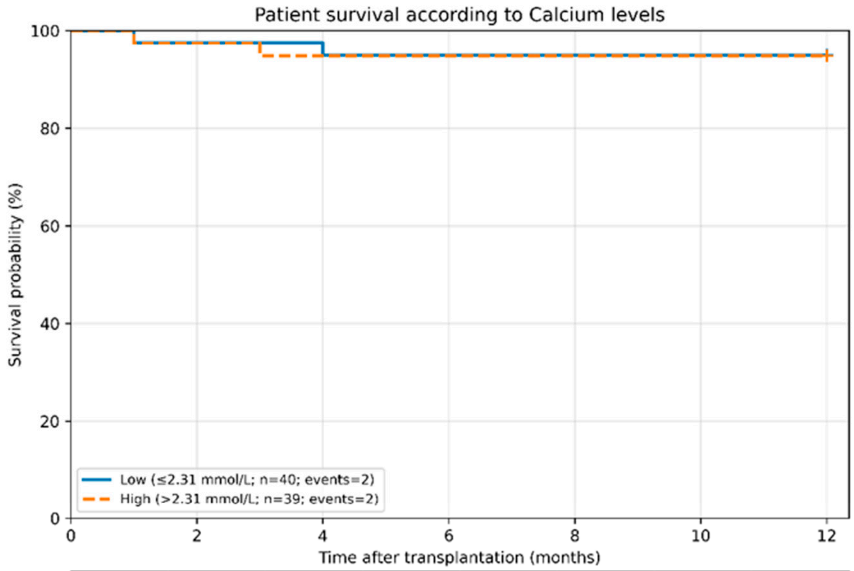

Number at risk	0	2	4	6	8	10	12
Low	40	39	39	38	38	38	38
High	39	38	37	37	37	37	37

Log-rank: χ^2=0.001, p=0.974. HR (High vs Low; Low reference)=1.033, 95% CI 0.145–7.333; Cox p=0.974. Tick marks indicate censoring; marks at 12 months overlap.

Figure S4. Kaplan-Meier survival curve for patient survival in relation to serum level of calcium.

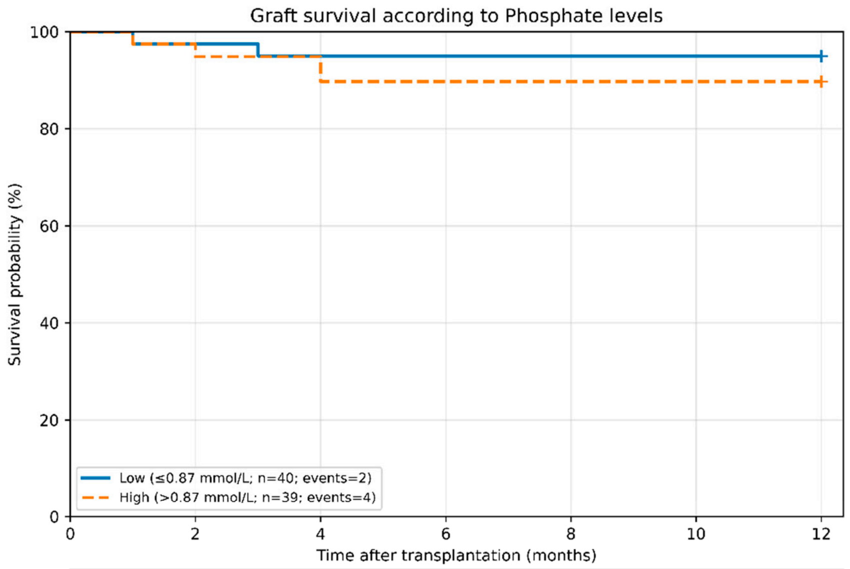

Number at risk	0	2	4	6	8	10	12
Low	40	39	38	38	38	38	38
High	39	38	37	35	35	35	35

Log-rank: χ^2=0.736, p=0.391. HR (High vs Low; Low reference)=2.071, 95% CI 0.379–11.309; Cox p=0.400. Tick marks indicate censoring; marks at 12 months overlap.

Figure S5. Kaplan-Meier survival curve for graft survival in relation to serum level of phosphate.

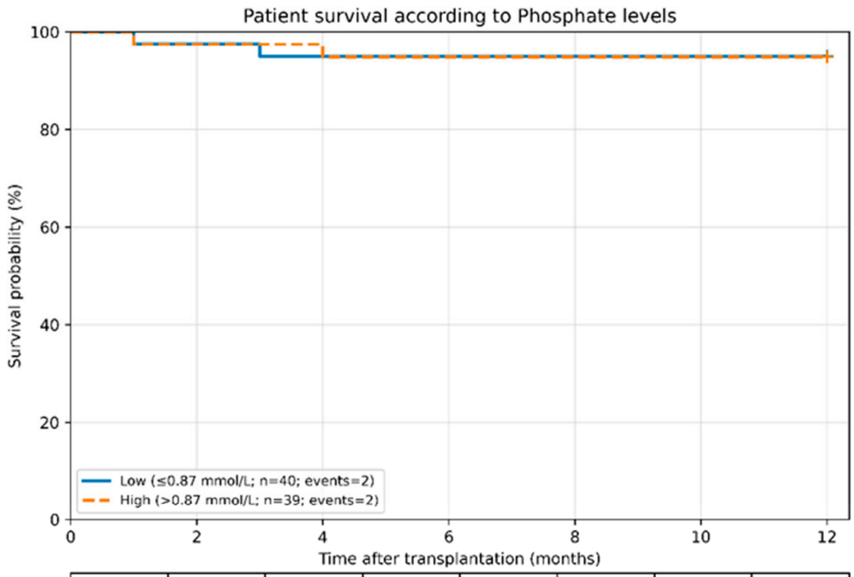

Number at risk	0	2	4	6	8	10	12
Low	40	39	38	38	38	38	38
High	39	38	38	37	37	37	37

Log-rank: χ^2=0.000, p=0.985. HR (High vs Low; Low reference)=1.019, 95% CI 0.144–7.237; Cox p=0.985. Tick marks indicate censoring; marks at 12 months overlap.

Figure S6. Kaplan-Meier survival curve for patient survival in relation to serum level of phosphate.

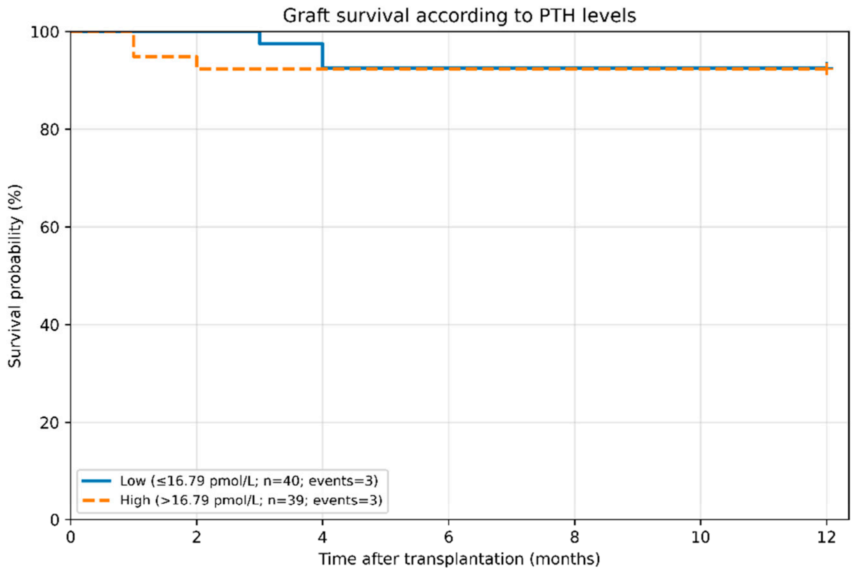

Number at risk	0	2	4	6	8	10	12
Low	40	40	39	37	37	37	37
High	39	37	36	36	36	36	36

Log-rank: χ^2=0.007, p=0.936. HR (High vs Low; Low reference)=1.068, 95% CI 0.216–5.292; Cox p=0.936. Tick marks indicate censoring; marks at 12 months overlap.

Figure S7. Kaplan-Meier survival curve for graft survival in relation to serum level of parathyroid hormone (PTH).

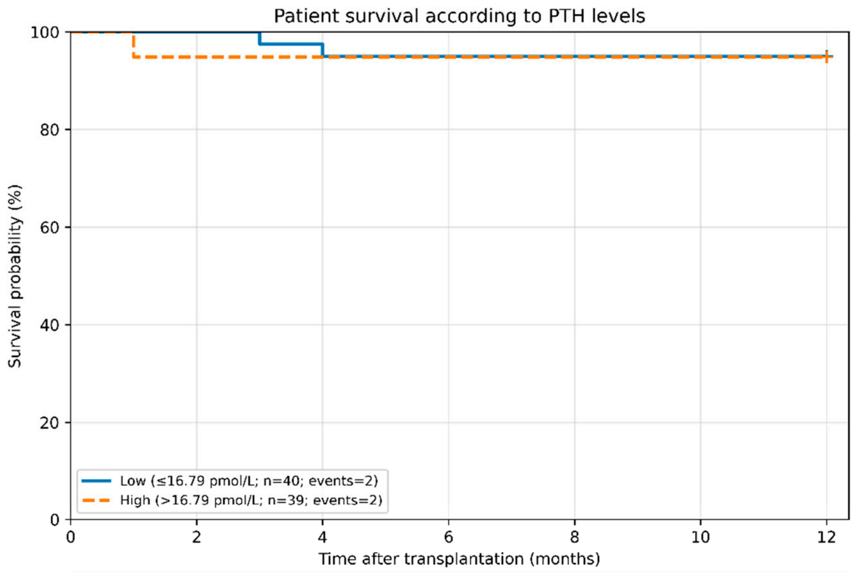

Number at risk	0	2	4	6	8	10	12
Low	40	40	39	38	38	38	38
High	39	37	37	37	37	37	37

Log-rank: χ^2=0.002, p=0.964. HR (High vs Low; Low reference)=1.053, 95% CI 0.148–7.477; Cox p=0.959. Tick marks indicate censoring; marks at 12 months overlap.

Figure S8. Kaplan-Meier survival curve for patient survival in relation to serum level of parathyroid hormone (PTH).
